# Computational identification of Y-linked markers and genes in the grass carp genome by using a pool-and-sequence method

**DOI:** 10.1038/s41598-017-08476-y

**Published:** 2017-08-15

**Authors:** Aidi Zhang, Rong Huang, Liangming Chen, Lv Xiong, Libo He, Yongming Li, Lanjie Liao, Zuoyan Zhu, Yaping Wang

**Affiliations:** 1 State Key Laboratory of Freshwater Ecology and Biotechnology, Institute of Hydrobiology, Chinese Academy of Sciences, Wuhan, 430072 China; 2 University of Chinese Academy of Sciences, Beijing, 100049 China

## Abstract

The molecular analysis of sex in vertebrates is important, as it has the potential to provide vital information for theoretical and applied research alike. Teleost fish are the ancient vertebrates that present a broad sex chromosome system but lack differentiated sex chromosomes in most species. Hence understanding the sex in fish would not only illuminate the sex determination evolution in vertebrates but also shed light on fish farming. In the present study, we used grass carp as a teleost fish model, studied the Y chromosome by using a pool-and-sequence strategy in combination with fragment-ratio method. In total, we identified five Y-linked scaffolds (totaling 347 Kb) and six Y-specific sequences that could be used as sex-specific markers, demonstrating the suitability of NGS-based re-sequencing of pooled DNAs for the identification of sex markers in fish. Moreover, 14 putative Y-linked genes were described for the first time. All the genes, except for *un-y1*, *un-y2*, and *ubq-y*, showed high similarity to their female homologs. RT-PCR revealed that *ubq-y* was only expressed in the male hypothalamus and pituitary. These findings provided an abundant resource for the Y chromosome of grass carp, and may help elucidate sex chromosome evolution in cyprinid fish.

## Introduction

Sex determination and differentiation are important issues in biology research. Teleost fish are the ancient vertebrates that present a broad sex chromosome system but lack differentiated sex chromosomes in most species. Hence, studying the evolution of sex chromosomes in teleost is challenging^1^. Understanding the sex determination mechanisms in fish would provide insights into the sex determination evolution in vertebrates^2^. In many species, economic traits, such as growth rate, weight, and disease resistance, have been associated with sex^3^. Therefore, understanding the sex determination mechanisms in farmed fish is necessary. It is urgent to develop sex manipulation biotechnologies for sex control breeding^4, 5^.

In recent vertebrates and most fish species, sex is mainly determined by one or more genetic factors located in sex chromosomes or autosomes^6^. The two common sex chromosome systems in most species are XX/XY and ZZ/ZW. However, some species present other types, such as XX/XO type in Giant freshwater stingray (*Himantura dalyensis*)^7^, X1X1X2X2/X1X2Y type in Neotropical gymnotiformes electric fish (*Gymnotiformes*)^8^, ZZ/ZW1W2 type in Apareiodon hasemani eigenmann (*Characiformes: Parodontidae*)^9^ and so on^10^. Compared with recent vertebrates, fish lack morphologically and genetically differentiated sex chromosomes. Moreover, there are frequent recombinations between X chromosome and Y chromosome. Until now, the studies that focused on sex-specific regions and genes in fish remain rare, thus the sex-specific markers and sex-related genes must be identified to elucidate the complicated mechanisms underlying various sex determination types.

Traditionally, fish geneticists adopt multiple molecular marker technologies to screen sex-specific DNA markers, such as amplified fragment length polymorphic DNA, single nucleotide polymorphisms, random amplified polymorphic DNA, simple sequence repeats and quantitative trait locus^11–14^. These DNA markers could help locate sex-related genes. *DM-related Y-specific gene (dmy)*, the representative sex-determining gene, was identified in Medaka (*Oryzias latipes*) through the traditional strategy^15, 16^. However, traditional experiments is expensive and time consuming, its screening scale is fairly limited. Therefore, the efficiency of identifying sex-specific markers could still be improved. The advent of high-throughput data and the development of bioinformatics methods have made identification of sex-specific region possible at a genomic scale. Y-linked regions and genes are being screened in more and more species recently^17–20^. Hall *et al*. identified six novel Y-linked genes in Mosquitos (*Aedes aegypti*) through Illumina sequencing of the male and female genomes basing on previously assembled genomes, one distant homolog of transformer-2 gene, named *nix*, was screened and proven to be the male-determining gene in Mosquitos^17, 21^. Meanwhile, Bidon *et al*. compared two *in silico* approaches and found that the method based on differences in the average read depth of autosomal versus sex chromosomal scaffolds was more efficient than that was based on similarity searching of known Y-linked genes^19^. With the former method, a 1.9 Mb region of Y chromosome was identified from the Polar bear (*Ursus groenlandicus*) genome. These studies demonstrated the reliability of the sequencing read depth approach that sequenced the pools of male and female individuals separately. As a result, the region wherein only male reads are aligned is regarded as the candidate sequence for the Y chromosome. However, the above approaches must be based on previous male genome assemblies.

Grass carp (*Ctenopharyngodon idellus*) is an important freshwater aquaculture cyprinid fish worldwide, and its genome has been recently sequenced through whole-genome shotgun sequencing in a gynogenetic female adult and a wild water-captured male adult grass carp respectively^22^. Comparative analysis of the zebrafish genome revealed that the LG24 linkage group of grass carp corresponds to chromosomes 10 and 22 of zebrafish, suggesting that the grass carp genome underwent chromosome fusion during evolution. Therefore, grass carp is an interesting model to study the evolution of sex chromosomes in fish. Our previous work adopted comparison of assemblies between one male and one female, and found one out of 206 contigs that demonstrated male specific amplification finally. The efficiency is still relatively low that may result from individuals’ difference. Hence in the present study, basing on our previous male grass carp genome data, we tested the suitability and reliability of the sequencing read depth approach of pooled DNAs in the identification of sex markers in fish. Finally, we computationally identified 347 Kb Y-linked sequences and 14 Y-linked genes. Six Y-linked sequences are unique in males, demonstrating a higher efficiency. Moreover, one Y-linked gene *ubiquitin ligase gene* (*ubq-y*) presents a male-specific expression, further studies of this gene may help elucidate its roles in sex determination and differentiation. Our findings will provide valuable insights into the evolution of fish sex chromosome.

## Results

### Sexual proportion statistics

**Figure 1:**
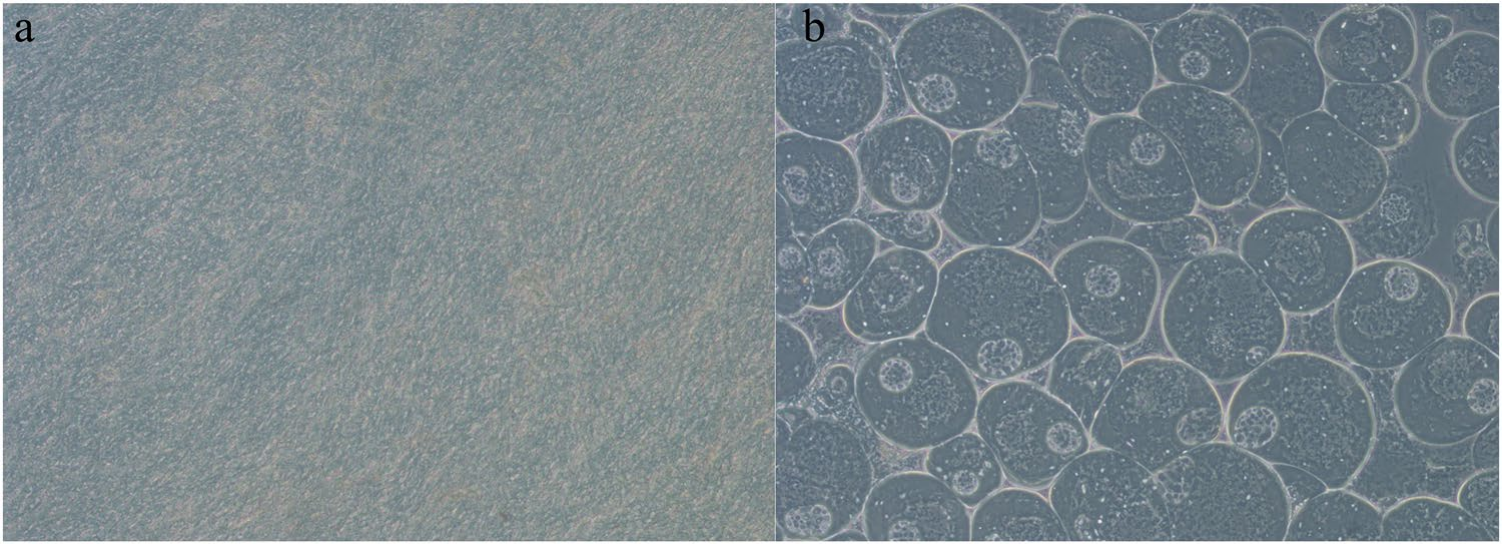
Micrographs of testis and ovary from 18-month-old grass carp. (**a**) Micrograph of testis in 10× magnification. (**b**) Micrograph of ovary gonad in 10× magnification.

A total of 90 grass carp individuals (18 months old) were selected randomly from the full-sib population and dissected to observe the gonad development under the microscope. Micrographs of the gonads are shown in Fig. 1. The result of gender identification revealed a sex ratio close to 1:1 with 47 females and 43 males. Moreover, the 90 grass carp individuals from the gynogenetic population were all females. The sexual proportion statistics confirmed that grass carp has a heteromorphic XX/XY sex chromosome system. Females, featuring two X chromosomes, are the homogametic sex, whereas males, featuring one X and Y chromosome each, are the heterogametic sex.

### Preliminary data analysis of re-sequencing of pooled DNAs

A total of 30 female and 30 male grass carp individuals at 18 months old were selected from the full-sib population. DNA was extracted, and two DNA mixed pools were constructed, hereafter referred to as female-pool and male-pool. The male-gc-assembly were softmasked using RepeatMasker and split using the repeat region as a separator. Consequently, 605,448 fragments were retained with a size of 749 Mb and an N50 value of 1,760 bp. The sizes of clean data from the female-pool and the male-pool were 33.33 Gb and 32.93 Gb, including 222,208,685 and 219,531,391 reads, respectively. The statistics of reference-guided mapping is shown in Table 1. Although the number of total reads from the female-pool was higher than that from the male-pool, the mapped reads (153,321,131) of the former was less than that of the latter (159,341,024), and mapping rates corresponded to 69% and 72.58%, respectively. The higher mapping rate of the male-pool suggested the existence of male-specific sequences in the reference that cannot be sequenced in the female-pool. The overall process for identifying Y-linked sequences and genes in the grass carp genome is shown in Supplementary Fig. S1.

**Table 1:** Summary of sequence data from the pooled libraries.

Samples	Total reads	Mapped reads	Mapping rate (%)	Average length	Insert size average	Average depth (X)	Coverage at least 1X (%)	Coverage at least 4X (%)
female-pool	222,208,685	153,321,131	69.00%	150 bp	364 bp	24.68 X	92.58%	75.16%
male-pool	219,531,391	159,341,024	72.58%	150 bp	360 bp	25.08 X	94.20%	80.12%

### Identification of putative Y-linked scaffolds

The fragment-ratio method was applied to identify Y-linked fragments. We introduced several abbreviations in this method as shown in Materials and Methods. F_i_-reads is the number of female-pool aligned reads against Fragment_i_ in male-gc-assembly, and M_i_-reads is the number of male-pool aligned reads against Fragment_i_ in male-gc-assembly. Thus, R_i_ denotes the ratio that divides the F_i_-reads by the M_i_-reads, R_i_-norm is the normalization of R_i_ value, and can fairly describe the difference in the ratio of alignment reads against the reference genome between the female-pool and the male-pool. The average R_i_-norm for all the fragments was 0.905 (Supplementary Fig. S2). We set a threshold of R_i_-norm <0.3 to differentiate Y-linked fragments from autosome and X fragments. Considering that false positives could result from low coverage, we excluded fragments with M_i_-reads less than 15. Finally, 923 fragments from 0 to 0.3 were considered as putative Y-linked fragments, involving 107 scaffolds with a size of 1,121 Kb. Of the 923 fragments, 169 have R_i_-norm values below 0.15. Information for these fragments is shown in the Supplementary Table S1.

**Figure 2:**
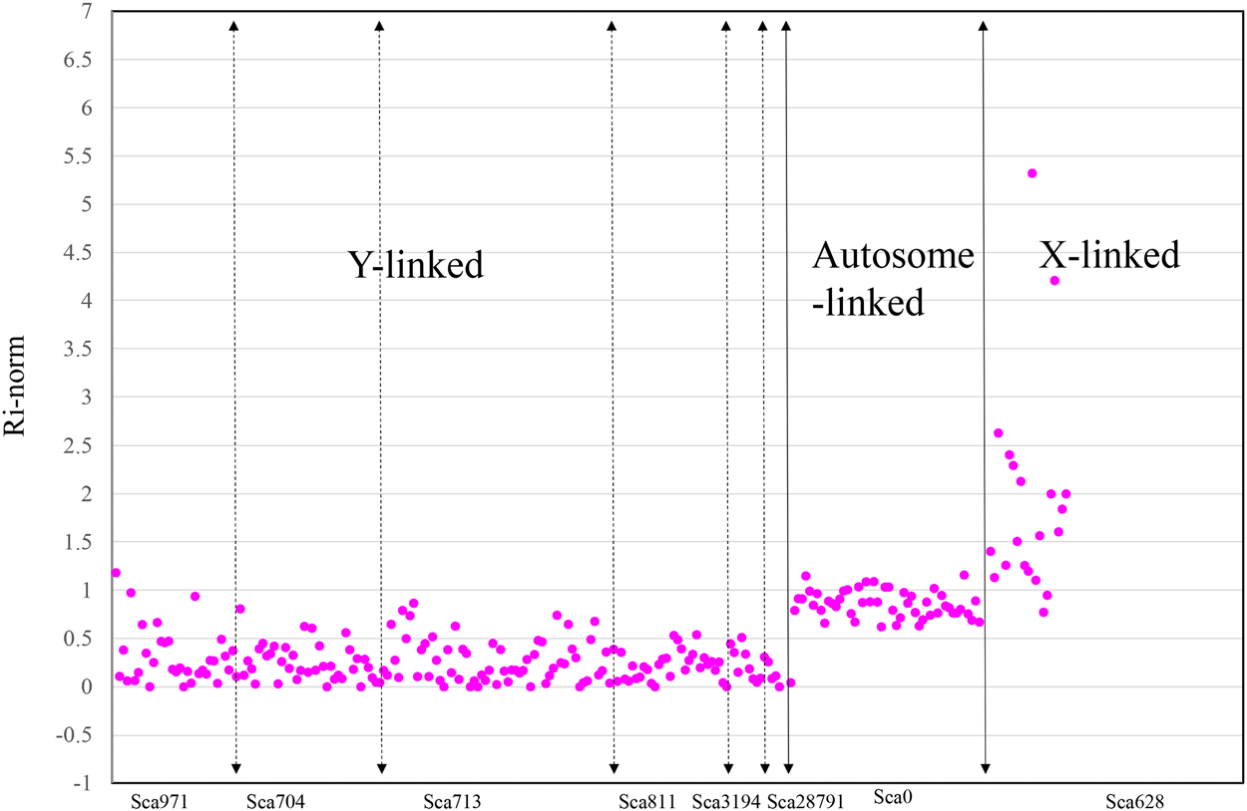
Discrepancy of R_i_-norm distribution among fragments that originated from different chromosomes. Pink circle indicates R_i_-norm values of fragments. The x axis represents different fragments, and the y axis represents R_i_-norm value. Scaffolds are shown by their ID numbers. Compared with autosome-linked scaffolds that with R_i_-norm value of ~1, Y-linked scaffolds have lower R_i_-norm value (~0), whereas X-linked scaffolds have higher R_i_-norm value (~2).

Since scaffolds from Y chromosome is composed of a number of Y-linked fragments, then it is reasonable that scaffolds from Y chromosome tend to have higher rate of Y-linked fragments when compared with scaffolds from autosome. Hence, an enrichment analysis was performed on the 107 scaffolds following the formula that were described in Materials and Methods. Only the *P*-values below 1e-005 were supposed to be significantly enriched. Finally, six out of 107 scaffolds were detected to have significantly more fragments with R_i_-norm values less than 0.3 (*P*-value < 1e-005). These six scaffolds are Sca3194, Sca704, Sca713, Sca28791, Sca811, and Sca971 (Table 2). Take Sca971 for example, the length of Sca971 is about 45,330 bp, a total of 28 fragments were produced, interestingly, 18 out of 28 fragments have the R_i_-norm values below 0.3, while 10 fragments have the R_i_-norm values below 0.15, the *P*-value of Sca971 is 2.166655e-44. Conversely, for putative autosome-linked scaffolds, such as Sca667, rare putative Y-linked fragments (2/78) were detected, and its *P*-value was 0.006456602 that above 1e-005. The discrepancies in R_i_-norm distribution for scaffolds that originated from different chromosomes are shown in Fig. 2. Compared with autosome-linked scaffolds (Sca0), putative Y-linked scaffolds have a lower R_i_-norm value (~0). Conversely, putative X-linked scaffolds (Sca628) have a higher R_i_-norm value (~2). Thus, the six putative scaffolds, with a combined size of 356 Kb, were more likely to be from the Y chromosome.

**Table 2:** Enrichment analysis of Y-linked scaffolds using hypergeometric test in R.

Chromosome	Scaffold	Length (bp)	Total number of fragments (*M*)	Number of fragments (R_i_-norm < 0.3)(*k*)	*P-*value (R_i_-norm < 0.3)
Y-linked scaffolds	Sca971	45,330	28	18	2.166655e-44
	Sca704	113,872	48	31	1.183675e-75
	Sca713	110,109	52	30	5.078515e-71
	Sca811	76,694	41	28	1.534825e-69
	Sca3194	8,996	7	6	8.634194e-17
	Sca28791	1,242	2	2	2.321559e-06
Autosomal-linked scaffold (control)	Sca667	136,669	78	2	6.4566e-3
	Sca641	143,109	58	2	3.626282e-3

Note: Number of total fragments in the genome reference (*N*) is 605,448; Number of total number of fragments (R_i_-norm < 0.3) in the genome reference (*n*) is 923. Putative autosome scaffolds are used as controls.

### High abundance of repetitive elements on the Y-linked scaffolds

For the six putative Y-linked scaffolds, we analyzed their repeat contents. Compared with whole male genome, 17.53% of the bases were masked as repetitive elements, the repetitive elements rate of Y-linked scaffold reached up to 30.71%. The detailed information for the repetitive elements and gaps is listed in Supplementary Tables S3 and S4. Moreover, we found a remarkably higher abundance of retro elements on the Y-linked scaffolds. The coverage of the retro elements is nearly five times as much sequences compared with the entire genome (17.16% vs. 3.53%). Additionally, the average coverage of long interspersed nuclear elements on the Y-linked scaffolds was 10.43%, which is 10 times more than that of the whole genome (1.34%). Furthermore, more long-terminal-repeat elements, L2/CR1/Rex, interspersed and simple repeats were detected in the Y-linked scaffolds. Notwithstanding, similar rates of DNA transposons (10.41% vs. 12.03%) were present in both Y-linked scaffolds and entire genome.

### PCR tests in of full-sib population confirmed Y-linked sequences

**Figure 3:**
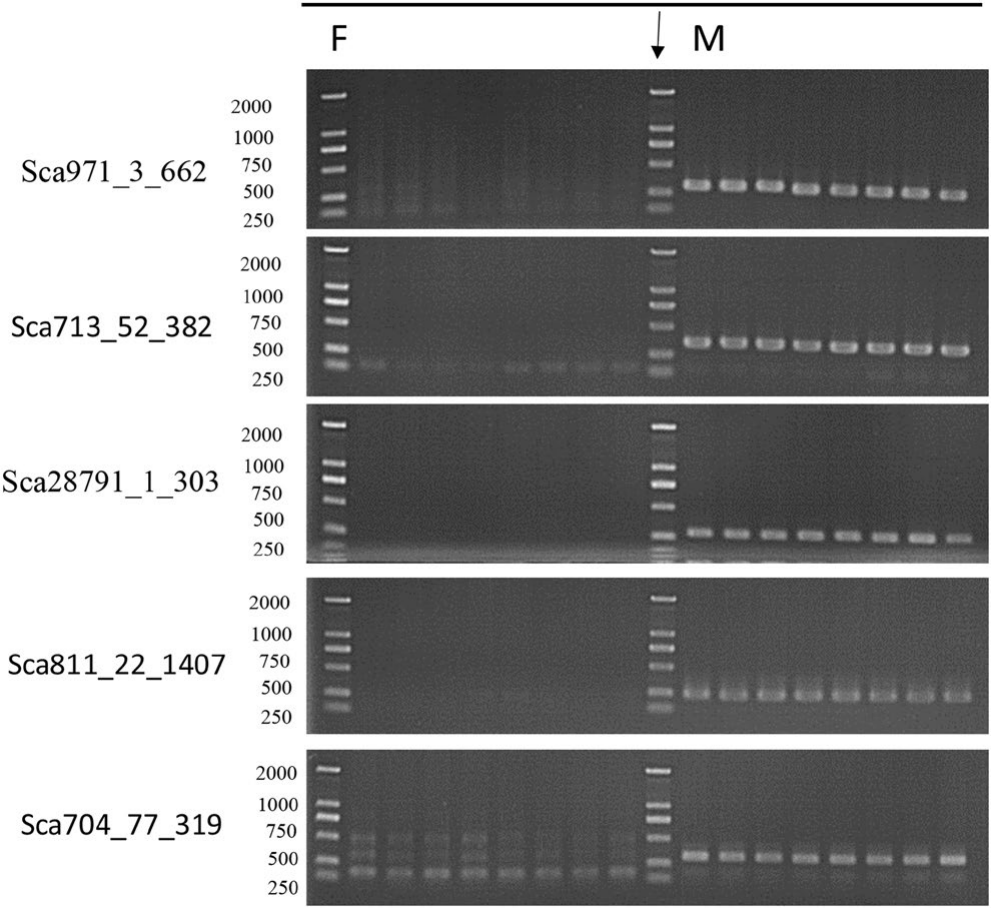
PCR tests for five representative Y-linked fragments in full-sib grass carp individuals. All five fragments that distributed in five significantly enriched scaffolds (Sca971, Sca811, Sca713, Sca28791, and Sca704) were selected in PCR tests in eight female and male grass carp samples. These five sequences are Sca971_3_662, Sca713_52_382, Sca28791_1_303, Sca811_22_1407, Sca704_77_319. Male specificity was defined as the occurrence of a clear amplicon of a distinct size in males but not in females. The DL2000 DNA Markers are loaded in the Lanes. The result verified that these five putative Y-linked scaffolds were Y-linked. Additional information of these five representative Y-linked fragments can be found in Table 3.

We selected a batch of putative Y-linked sequences from the six scaffolds for further PCR verification in the full-sib population. To improve the efficiency of this process, we chose fragments that have higher stringent threshold (R_i_-norm < 0.15). Thus, 75 fragments were included for subsequent analysis. A BlASTN search^23^ was performed against female-gc-assembly using default parameters^22^, and fragments with best blast hits (expect value < 1e-005) were discarded. Finally, 17 sequences across five scaffolds (except Sca3194) were retained in PCR amplification in the full-sib population. The detailed information is listed in Supplementary Table S2. PCR amplification was performed to test the accuracy of our approach. Fragments were defined as Y-linked when a single, clear amplification product was detected in males, whereas no amplicons or only low-intensity bands of different sizes were observed in females. Full-sib PCR results showed that most fragments with R_i_-norm less than 0.15 were amplified only in males with an accuracy of 94.12% (16/17) (Supplementary Table S2). Partial full-sib PCR results of representative sequences for the five scaffolds are shown in Table 3 and Fig. 3. Therefore, the five scaffolds were totally confirmed as Y-linked. The above results suggested that fragments with low R_i_-norm values tend to be Y chromosome sequences, and the fragment-ratio method is reliable for identifying Y-linked sequences in grass carp.

**Table 3:** Selected Y-linked fragments with R_i_-norm < 0.3, PCR results are shown in Fig. 3.

Fragment name	Length	F_i_-read	M_i_-reads	R_i_-norm	PCR (full-sib)	PCR (wild)
Sca971_3_662	662	0	34	0	Y	Y
Sca713_52_382	382	1	45	0.022	Y	N
Sca28791_1_303	303	2	47	0.043	Y	N
Sca811_22_1407	1407	2	58	0.034	Y	N
Sca704_77_319	319	0	11	0	Y	N

Note: F_i_-reads is the number of female-pool aligned reads against Fragment_i_ in male-gc-assembly, M_i_-reads is the number of male-pool aligned reads against Fragment_i_ in male-gc-assembly. Thus, R_i_ denotes the ratio that divides the F_i_-reads by the Mi-reads, R_i_-norm is the normalization of R_i_ value, and can fairly describe the difference in the ratio of alignment reads against the reference genome between the female-pool and the male-pool. In the seventh and eighth columns, Y represents the corresponding sequence that was only amplified successfully in male individuals, and N represents the corresponding sequence that was amplified in both male and female individuals.

### PCR tests in wild population confirmed Y-specific sequences

**Figure 4:**
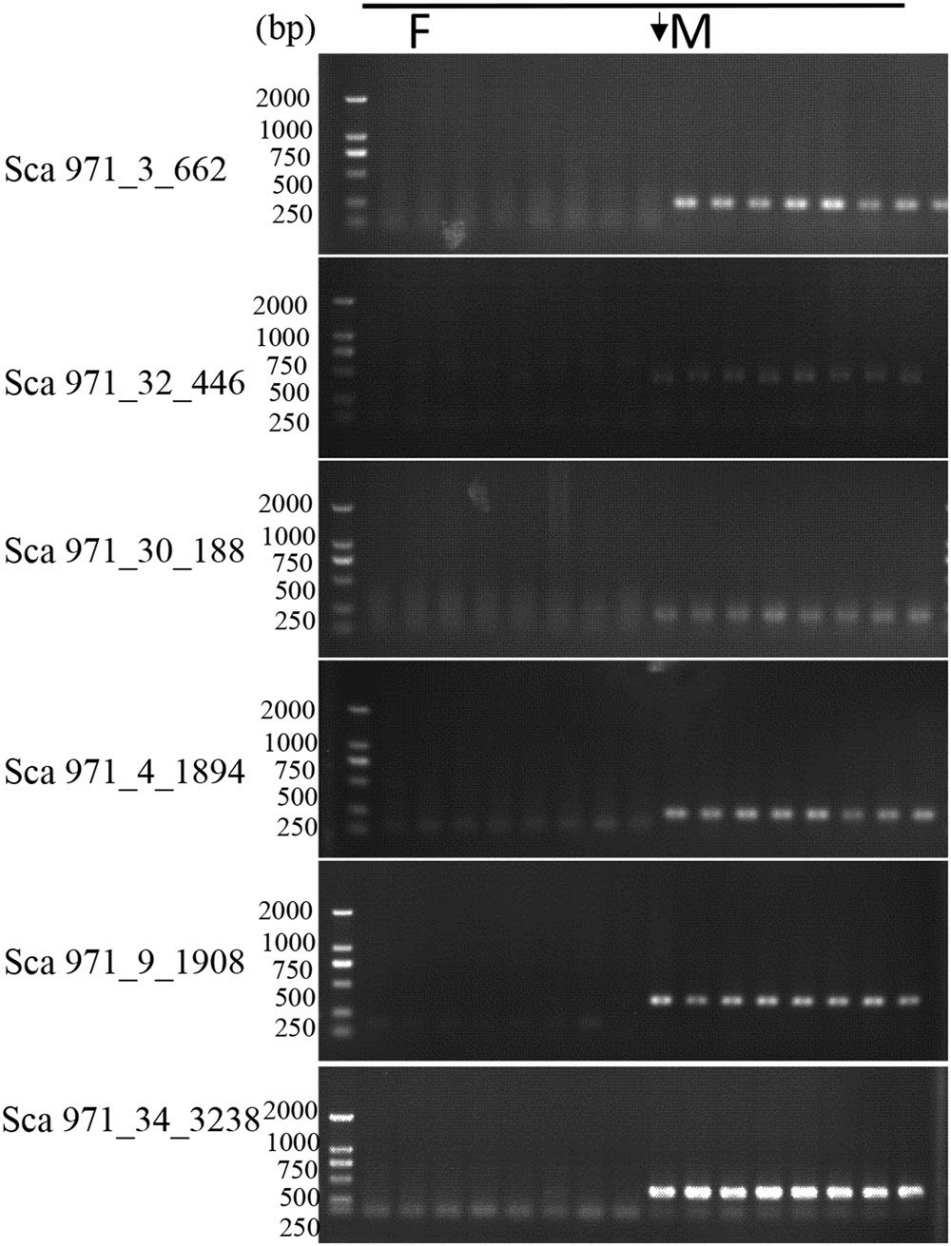
PCR tests for fragments that distributed in Sca971 in wild grass carp individuals. Male specificity was defined as the occurrence of a clear amplicon of a distinct size in males but not in females. The DL2000 DNA Markers are loaded in the Lanes. The results showed that all the six sequences of Sca971 were only amplified in wild male grass carps.

Differences in the genetic background among different families may cause sequence divergence for the Y chromosome and affect test efficiency. Therefore, eight female and eight male wild grass carp individuals from different water systems (Zhujiang river, Yangtse river, Xiangjiang river, Lao river.) that were described in our previous study^24^, were collected. The detection of polymorphism in those wild grass carps confirmed that the natural germplasm resources of wild grass carp were genetically diverse^24^. Further PCR tests were performed in these wild grass carps to confirm that whether the Y-linked sequence is specific to wild male individuals. However, the results of further PCR in wild grass carps were below our expectations (Supplementary Fig. S3). All six sequences of Sca971 (Sca971_3_662, Sca971_30_188, Sca971_4_1894, Sca971_9_1908, and Sca971_32_446, Sca971_34_3238) still presented male-specific amplification (see Fig. 4). These sequences have the potential to be used as sex-specific molecular markers. By contrast, the 11 other sequences (Sca 704_15_1909, Sca 704_33_2332, Sca 704_63_267, Sca 704_77_319, Sca 713_22_716, Sca 713_52_382, Sca 713_68_619, Sca 713_93_3485, Sca 811_22_1407, Sca 811_50_718, Sca 28791_1_303) from other scaffolds could be amplified in certain wild females, as shown in Supplementary Table S2.

### Y-linked genes annotation

**Figure 5:**
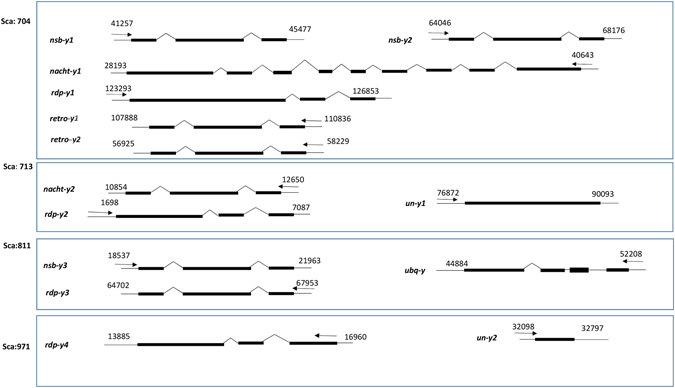
Predicted gene models of Y-linked scaffolds (Sca971, Sca811, Sca713, and Sca704). Numbers above gene structures indicate predicted start/end position of protein translation in the scaffold. Directions of translation are indicated by black arrowheads. Black boxes with different length represent predicted exons of gene.

Identification of Y-linked sequences is important to elucidate the gene content of Y chromosomes. The five confirmed Y-linked scaffolds (Sca704, Sca713, Sca28791, Sca811, and Sca971) were used for gene annotation based on BLAST evidence. Alignments detected in at least one database (NR, Ref-Seq, EST) were considered Y-linked genes. Thus, four scaffolds presented hits in at least one database. A total of 14 genes were annotated from BLAST evidence and were named according to their putative functions or as ‘*un-y’* for the genes without previously described functions. Detailed information of all Y-linked genes is listed in Table 4 and Fig. 5. We identified three copies of *neoverrucotoxin subunit beta gene* (*nsb-y1*, *nsb-y2*, and *nsb-y3*) that located in Sca704 and Sca811. These genes contain Fibronectin type-III, neoverrucotoxin subunit domains and were predicted to play roles in microtubule organization and stabilization^25^. Furthermore, two copies of the *NACHT, LRR and PYD domains-containing gene* (*nacht-y1* and *nacht-y2*) were identified in Sca704 and Sca713, they were supposed to function in innate immunity and inflammation^26^. *RNA-directed DNA polymerase genes* (*rdp-y1*, *rdp-y2*, *rdp-y3*, and *rdp-y4*) were widespread in all of these four scaffolds and may play roles in DNA replication and respond to genotoxic stress^27^. Two copies of *retrotransposon-like gene* (*retro-y1* and *retro-y2*) were identified in Sca704^28^. One gene that supposed to be an E3 ubiquitin ligase containing a Zinc finger domain^29^, named *ubq-y* gene, was identified in Sca811. All the above genes had Ref-Seq evidence and shared homology with proteins with known functions. In addition, nucleotide BLAST results against the grass carp annotated genes showed that all the genes have female homologs with high sequence identities except *ubq-y* gene. The coding sequence of *ubq-y* is obviously divergent from its female homolog. Two other genes were detected in Sca713 and Sca971, nucleotide BLAST results showed that they only have EST evidences and share no similarity to proteins with known functions. We named them *unknown function genes* (*un-y1, un-y2*), these two genes could be novel genes because of their lack of similarity to known function genes. The BLAST results showed that many genes are incomplete and some of them lack exons, it is understandable because Y-linked sequences tend to have sequence assembly errors resulting from highly repetitive sequences scattered in the Y chromosome. Therefore, re-sequencing and rapid amplification of complementary DNA are usually needed to fully annotate Y-linked genes.

**Table 4:** Y-linked gene annotation.

Gene symbol	Scaffold	Ref-Seq (EST) homolog	Amino acid identity with ref	Female-gc-assembly homolog	Domain	Putative function
*nsb-y1*	Sca704	XP_016118179.1^47^	76%	Yes	Fibronectin type-III, SPRY domains	Microtubule organization and stabilization^25^
*nsb -Y2*	Sca811					
*nsb -Y3*						
*nacht-y1*	Sca704	XP_016362555.1^47^	69%	Yes	NACHT, LRR and PYD domains	Innate immunity and inflammation^26^
*nacht-y2*	Sca713					
*rdp-y1*	Sca704	XP_014000594.1^48^	71%	Yes	Rve,	RNA-directed DNA polymerase^27^
					RVT_1	
*rdp-y2*	Sca713					
*rdp-y3*	Sca811					
*rdp-y4* ^a^	Sca971					
*retro-y1 retro-y2*	Sca704	XP_007859699^49^	74%	Yes	Undefined	Development of the placenta^28^
*un-y1* ^a^	Sca713	CT602791.2	84%	No	Undefined	Undefined
*ubq-y* ^a^	Sca811	XP_016405694.1	98%	Yes	IBR	Probable E3 ubiquitin-protein ligase^29^
		GW811780.1^50^ EE398743.1	93%			
*un-y2* ^a^	Sca971	GT224642.1^50^ GR943694.1 GW812769.1 DT343035.1 EG534739.1	95%	No	Undefined	Undefined

Notes: In the cases where the homolog is an annotated gene, we used the homolog gene name as an identifier, otherwise we used the “*un-y*” as an identifier. Amino acid identity with ref were computed by comparing sequences directly.

^a^Genes in bold were selected for RT-PCR experiments, as shown in Fig. 6.

### Expression analysis of four Y-linked genes

**Figure 6:**
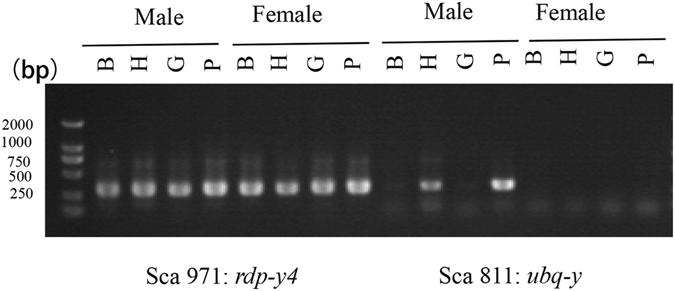
Expression of two Y-linked genes (*rdp-y4, ubq-y*) in females and males. Template cDNAs were derived from four tissues (B: brain, H: hypothalamus, G: gonad, and P: pituitary). Each tissue was extracted from three adult male/female individuals. *rdp-y4* gene was amplified in all four tissues in both sexes with high abundance, thereby confirming the quality of the cDNA templates. *ubq-y* was not detected in the female tissues and was highly expressed in two male tissues (hypothalamus and pituitary).

Most Y-linked genes showed high similarity to female homologs, testing male-specific expression is difficult owing to the high homologous sequence. Hence, we only chose four genes, including three genes (*ubq-y, un-y1, un-y2*) that lack highly similar female homologs, and one gene *rdp-y4* that have highly similar female homolog. Expression experiments were performed in four female and male tissues (B: brain, H: hypothalamus, G: gonad, P: pituitary). The result is shown in Fig. 6. We found that *rdp-y4* gene was amplified in all four tissues in both sexes with high abundance, thereby confirming the quality of the cDNA template. Hence, *rdp-y4* can be used as the positive control. Noticeably, the *ubq-y* mRNA was highly expressed in two male tissues (hypothalamus and pituitary), but not detected in the female tissues. Expression specificity in male neuroendocrine tissues indicated that *ubq-y* may function in sex differentiation. By contrast, the *un-y1* and *un-y2* genes were not amplified in both sexes, suggesting that they may be expressed during developmental stages, such as embryonic or nymphal stage.

**Figure 7:**
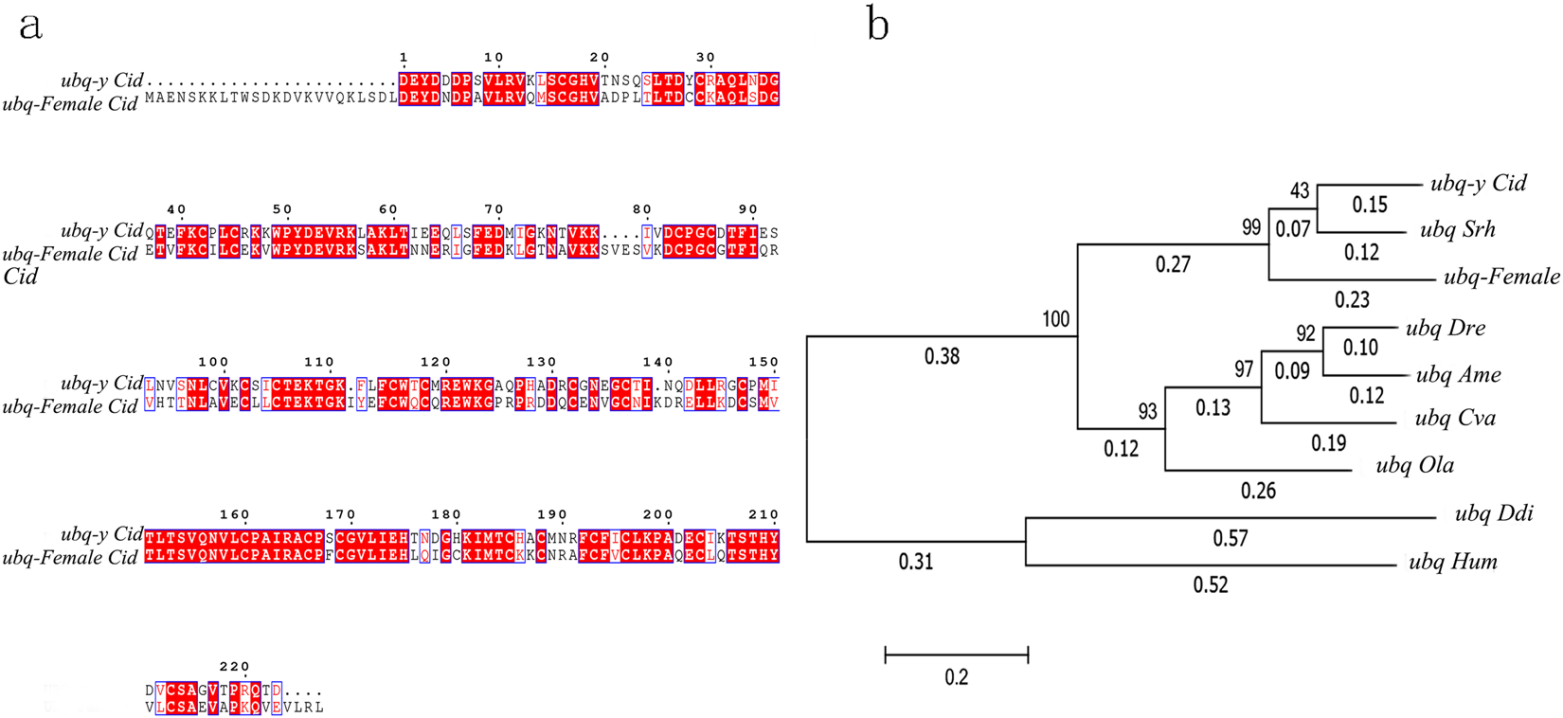
Phylogenetic analysis of *ubq* genes and alignment of grass carp *ubq-y* gene with its female homolog. (**a**) Alignment of coding protein sequences between *ubq-y* gene and its female homolog in grass carp. Sequences were aligned with ClustalW2. The result showed widespread differences in amino acid sites, suggesting that *ubq-y* gene is a divergent paralog (amino acid identity of 75%). (**b**) Phylogenetic analysis of *ubq* genes. A Maximum Likelihood tree with Poisson correction was constructed using MEGA6^46^. Bootstrap support values (10,000 replicates) are shown inside the tree. The tree is drawn to scale, with branch lengths measured using the number of substitutions per site (below the branches). All positions with gaps and missing data have been eliminated. Sequence accession numbers are shown in Supplementary Table S7. Phylogenetic analyses of *ubq* genes suggested that *ubq-y* gene diverged from the female homologous gene in certain fish species. Species information are: *Sinocyclocheilus rhinocerous (Srh); Astyanax mexicanus (Ame); Cyprinodon variegatus (Cva); Oryzias latipes (Ola); Dictyostelium discoideum (Ddi); Homo sapiens (Hum); Danio rerio (Dre)*.

Considering that the *ubq-y* gene is only present in male grass carp, we compared its translated coding sequence with female homolog. Twenty four amino acids in N-terminal were failed to be predicted. Alignment results showed widespread discrepancies in amino acid sites (Fig. 7a), suggesting that *ubq-y* is a divergent paralog (amino acid identity of 75%). Phylogenetic analyses of *ubq-y* genes suggested that this gene diverged from its female homologous gene in certain fish species, such as *Sinocyclocheilus rhinocerous* (Fig. 7b), which is also a ray-finned fish in the *Cyprinidae* family. By contrast, no *ubq-y* ortholog was detected in zebrafish despite being in the same *Cyprinidae* family. These findings indicated that their divergence from zebrafish followed by independent divergence of the sex chromosomes in these two species. Both expression and sequence evidences revealed the divergent function of *ubq-y* gene.

## Discussion

The Y chromosome is involved in multiple biological functions, such as male fertility and sex determination. A male-determining gene in the Y chromosome is hypothesized to initiate male sexual differentiation, such as *Sex-determining region Y gene* (*sry*) in Human and *dmy* in Medaka^15, 30^. However, deciphering gene content and organization is difficult because of the highly heterochromatic nature and accumulation of repetitive DNA in sex chromosomes, especially the Y chromosome. Fish are known to have moderately differentiated sex chromosomes^31, 32^. Recombination events between the X and Y chromosomes are frequent, thereby complicating the characterization of sex-specific fish sequences and genes. Nevertheless, considering the diverse sex-determination types and evolutionary position, studying sex chromosomes in fish is valuable and important to evolutionary biology. Grass carp is an interesting model for sex chromosomes because of its sex chromosome fusion event described in our previous work^22^.

With the advent of high-throughput sequencing and reduced cost, various types of sequencing strategies were applied in acquisition of objective traits, including whole genome re-sequencing, restriction site associated DNA sequencing (RAD-seq), exome sequencing^33, 34^ and so on. Despite of higher cost, whole genome re-sequencing (WGS) demonstrates its advantage of higher coverage compared with other next-generation sequencing (NGS) technologies. Whole genome re-sequencing of pools (Pool-seq) is becoming an efficient and economical way to acquire the objective traits, and applied in identifying markers linked to any specific gene or genomic region^35^. In our study, we performed whole genome re-sequencing of pooled gDNA samples of both sexes in grass carp. A fragment-ratio method was successfully applied to identify five Y-linked scaffolds (totaling 347 Kb), with a 94.12% accuracy of PCR tests in full-sibs (16/17). This method is based on the discrepancy in the ratio of alignment reads against the male reference genome between the female-pool and the male-pool. Thus, it could work in large and complex genomes and can be applied to any species with differentiated sex chromosomes. Coincidentally, numerous Y-linked genes have been successfully described following the same principle in various species, such as the Kissing bug (*Triatoma dimidiata*), Polar bear, and Mosquitos^17–19^. Our study further confirmed the efficacy of such comparative genomic strategies in fish. Compared with traditional methods, such as AFLP and SNP, these comparative genomic strategies are more efficient and time saving, especially in detecting large Y-linked region and genes.

However, the species previously studied have highly evolved gonosomes, and the prominent divergence between sex chromosomes make it very easy to acquire Y-specific sequences and genes. In contrast, identifying Y-specific genes in fish is complicated by their primitive property accompanied by the high sequence similarity of gonosomes. Although the method we used follows the same principle of the CQ method proposed by Hall *et al*.^32, 36^, it has a higher rate of false positivity in PCR tests in the wild grass carp population compared with Mosquitoes. Only 6 out of 17 Y-linked sequences were proven male specific in the wild grass carp population. The undifferentiated status of fish gonosomes and high frequent crossovers between X and Y chromosome likely explain much of this phenomenon. For Y chromosome, the high similarity to X chromosome counterpart make it difficult to distinguish from each other through a pair of primer. Our results indicated the crucial influence affected by discrepancy of gonosomes. The higher the similarity between X and Y chromosome, the lower the chance of finding true Y-specific sequences and genes. Notwithstanding, we still screened out six Y-specific sequences (spanning 48Kb) that could be used as sex-specific markers with a higher efficiency compared with our previous work^22^, where only one out of 206 contigs retained its specificity in wild grass carps finally. Actually, the putative male-specific markers (probe 184, CI01M090293) that was described in our earlier publication^22^, was located on Sca971 around the 30 Kb position (30,957–34,697 bp), with a length of 4,148 bp. Additionally, the amplified product of the specific marker corresponds to Sca971_34_3238, which belongs to our six Y-specific sequences, was also verified by our PCR test in both full-sib and wild grass carp individuals (Supplementary Table S2.xls).

Placental mammals have highly evolved sex chromosomes, but lower recombination with other genome regions make the Y chromosome genetically degenerate with rare active genes^36^. Fish still have a primitive Y chromosome that evolved from recent evolutionary events, thus, its gene content is theoretically higher than that of placental mammals. Although we proved that the Y-linked scaffolds have a higher rate of repetitive elements compared with the whole genome (30.71% vs. 17.53%), 14 genes were still deciphered on the basis of homology searching. Except for *un-y1*, *un-y2*, and *ubq-y* genes, the Y-linked genes showed high similarities to their female homologs, most of them are multi-copy genes distributed in at least two loci. This phenomenon revealed that degeneration level of Y chromosome in fish is still primitive despite of accumulation of repetitive DNA. For Sca971, although we have confirmed the male specificity of six sequences, all of them spread across an intergenic region, the gene content of this scaffold still shares high sequence similarity to female homologs, such as *rdp-y4* gene. These findings indicated that the extent of divergence is variable across the region, with high similarity in coding sequences but low similarity in intergenic regions that result from mutation or indels with few functional constraints^37^.

We have no indication of the biological function of the two other Y-linked genes (*un-y1* and *un-y2*) because of the lack of similarity to genes with known functions. *ubq-y* gene was supposed to be an E3 ubiquitin-protein ligase containing a zinc finger domain and shared low sequence similarity to its female homolog. RT-PCR results further revealed that *ubq-y* is expressed only in the male hypothalamus and pituitary, suggesting *ubq-y* gene possibly participates in gender differentiation and development in grass carp. Future functional studies, such as RNAi silencing, are needed to clarify its biological role. The alignments and phylogenetic analysis showed the extensive sequence divergence of *ubq-y* with female homologs, suggesting the differentiation of sex chromosomes in certain cyprinid fish. Although the grass carp genome underwent whole-genome duplication similar to zebrafish after the teleost radiation, with its adaption to variable environments, grass carp undergone species-specific duplications involving cell proliferation and differentiation, as well as organ size control^22^, which make it higher in terms of sex differentiation. Grass carp already has incomplete differentiation of the Y chromosome compared with zebrafish, gene duplication and function divergences are accompanied by the evolution of sex chromosomes.

## Materials and Methods

### Ethical procedures

Animal welfare and experimental procedures were performed in accordance with the Guide for the Care and Use of Laboratory Animals (Ministry of Science and Technology of China, 2006), and the study protocol was approved by the Committee of the Institute of Hydrobiology, Chinese Academy of Sciences (CAS). All surgeries were performed under eugenol anesthesia, all efforts were made to minimize suffering.

### Construction of population and experimental fish collection

During the breeding season of grass carp and common carp (late April to early May), 1 female grass carp, 1 male grass carp, and 1 male common carp parents were randomly selected from the Guanqiao Experimental Station of the Institute of Hydrobiology, CAS (Wuhan, China). Semen or eggs from each fish were collected separately. A part of eggs were conventionally fertilized using grass carp semen to construct a full-sib population. Another part of eggs were fertilized using UV-inactivated semen of common carp and simulated in cold water at 0–4 °C to construct a gynogenetic population^38^. The two groups of fertilized eggs were hatched in different hatchery ponds for emerging fry. After 2 weeks, 500–800 fry with approximately 1 month old were selected randomly from each population and bred in two ponds approximately 2600 m^2^ in area.

At age of 18 months, weighing 800–1,200 g, with the average length of 30 cm, 90 grass carp individuals were selected randomly from each population and were dissected to observe gonad development under the microscope. In addition, eight female and eight male wild grass carp individuals were collected from different wild water systems (Zhujiang river, Yangtse river, Xiangjiang river, Lao river) that were described in our previous study. The GPS coordinates of the five water systems are listed in Supplementary Table S8. The detection of polymorphism in those wild grass carps confirmed that the natural germplasm resources of wild grass carp were genetically diverse^24^. Tail fins were cut from experimental fish and preserved in 95% ethanol.

The fish were fed in the Guan Qiao Experimental Station, Institute of Hydrobiology, Chinese Academy of Sciences, and acclimatized in aerated fresh water at 26–28 °C for one week before processing. Fish were fed with a commercial diet twice a day and water was exchanged daily.

### Re-sequencing of DNA pools of male and female grass carp

A total of 30 female and 30 male grass carp individuals at 18 months old were selected from the full-sib population. DNA was extracted from tail fins using the conventional phenol–chloroform method. Thus, two DNA mixed pools were constructed, hereafter referred to as female-pool and male-pool. DNA quality and purity were checked using 1% agarose gel electrophoresis and ultra-micro spectrophotometry (Nanodrop 2000, USA). A total of 1.5 μg DNA per sample was used as input material for the DNA library. Sequencing libraries were generated using TruSeqNano DNA HT Sample Preparation Kits (Illumina, USA) following the manufacturer’s protocol (NEBNext Ultra DNA Library Prep Kit for Illumina), index codes were added to attribute sequences to each sample. The DNA sample was disrupted via sonication into fragments of approximately 500 bp in size. The DNA fragments were end-polished, A-tailed, and ligated using the full-length adapter for Illumina sequencing with further PCR amplification. Finally, PCR products were purified, libraries were analyzed for size distribution using an Agilent2100 Bioanalyzer and quantified using real-time PCR. These libraries were sequenced using the Illumina HiSeq4000 platform, 150 bp paired-end reads were generated with an insert size of approximately 500 bp. Library constructions and sequencing were performed using Novogene Company (China). The sequencing data in this study have been deposited in the Sequence Read Archive (SRA) at the National Center for Biotechnology Information (NCBI) (accession number: SRP095438).

### Preliminary data analysis

The quality control of paired-end Illumina sequencing data was initially evaluated using the NGSQC Toolkit, and low-quality sequence data were filtered out (cutOffQualScore < 20)^39^. The grass carp genome assemblies were obtained from our previous work^22^. The female genome was referred to as female-gc-assembly, the male genome assembly that was utilized as the reference genome source was referred to as male-gc-assembly. Male-gc-assembly is arranged into 130,221 scaffolds with an N50 value of 2,269 Kb and a size of 1.07 Gb, its average sequence length is approximately 8,265 Kb, with the minimum and maximum at 246 bp and 16.34 Mb, respectively. The scaffolds in male-gc-assembly were labeled according to their respective scaffold ID numbers with the format “ScaID.” In consideration that repetitive sequences may result in a high bias of sequencing coverage, all scaffolds from male-gc-assembly were softmasked using RepeatMasker and split using the repeat region as a separator. Fragments with length shorter than 200 bp were disregarded.

RepeatMasker^40^ was used to softmask all scaffolds and avoid repetitive sequences (i.e., transposable elements, virus retrogenes, and simple repeats) using default parameters, except that the “-species” parameter was set as “Dario”. As a result, scaffolds in male-gc-assembly were fragmented into numerous fragments. Meanwhile, fragments shorter than 200 bp were excluded to avoid false positives. The remaining sequences were used as reference.

The treated cleaned reads were aligned to against the reference genome using the ultrafast read aligner bwa^41^. Samtools^42^ was used to index, merge, sort, remove, format convert, and remove duplications against the aligned data. Picard (http://picard.sourceforge.net/) was used to mark duplicated reads, and realignment of reads around regions of indels was performed using GATK^43^.

### Computational identification of putative Y-linked sequences

The fragment-ratio method was applied to identify Y-linked sequences, this method was based on the discrepancy in the ratio of alignment reads against the reference genome (male-gc-assembly) between the female-pool and the male-pool. For a given Fragment_i_, we can obtain R_i_-norm that was calculated using the following equations:

1 $${{\rm{R}}}_{{\rm{i}}}=({{\rm{F}}}_{{\rm{i}}} \mbox{-} \text{reads}/{{\rm{M}}}_{{\rm{i}}} \mbox{-} \text{reads})$$

2 $${\rm{norm}}=({{\rm{M}}}_{{\rm{total}}} \mbox{-} \text{reads}/{{\rm{F}}}_{{\rm{total}}} \mbox{-} \text{reads})$$

3 $${{\rm{R}}}_{{\rm{i}}}-{\rm{norm}}=({{\rm{F}}}_{{\rm{i}}} \mbox{-} \text{reads}/{{\rm{M}}}_{{\rm{i}}} \mbox{-} \text{reads})\ast \text{norm}$$

where F_i_-reads is the number of female-pool aligned reads against Fragment_i_ in male-gc-assembly; M_i_-reads is the number of male-pool aligned reads against Fragment_i_ in male-gc-assembly; R_i_ denotes the ratio that divides the F_i_-reads by the M_i_-reads. However, the discrepancy in the sequencing coverage of the two pools may affect the R_i_ value, leading to a biased result. Hence, R_i_ normalization was performed by multiplying by the value of norm, which denotes the normalized female-male ratio that was calculated by dividing F_total_-reads from M_total_-reads. Therefore, R_i_-norm can fairly describe the difference in the ratio of alignment reads against the reference genome between female-pool and male-pool. The depth statistics of all the fragments was calculated using the command “idxstats” in Samtools. Moreover, a program written in perl named “RatioOfSex.pl” was used to calculate the R_i_-norm value.

The fragment-ratio method is based on the difference in the relative numbers of X and Y chromosomes between females and males. Reads from the female-pool and the male-pool demonstrate the characteristics of sex-specific patterns when aligned against different chromosomes. For species with the XX/XY sex determination system, the female has two X chromosomes and no Y chromosome, whereas the male has one X and Y chromosome each. Moreover, both sexes carry two copies of each autosome. Thus, unique Y-linked sequences are not present in the female genome. On the basis of the above data, the values of R_i_-norm for Fragment_i_ vary from 0 to 2: ~0 for Y chromosome fragments, ~1 for autosome fragments, and ~2 for X chromosome fragments. Meanwhile, some Y-linked fragments have homologous regions to their X chromosome counterparts, leading to few aligned reads from the female-pool, hence R_i_-norm is greater than 0. We set a threshold of R_i_-norm as 0.3 to distinguish Y-linked sequences from autosomes and X chromosomes as previously described^17^. False positives could arise from low coverage, thus the resulting fragments with M_i_-reads less than 15 were excluded from further analysis.

Hypergeometric test in R was used to perform enrichment analysis for the Y-linked scaffolds following the below equation:

4 $$P=\sum _{i=0}^{k-1}\frac{(\begin{array}{c}M\\ i\end{array})(\begin{array}{c}N-M\\ n-i\end{array})}{(\begin{array}{c}N\\ n\end{array})}$$

where *N* and *n* are the total number of fragments and the total number of fragments with R_i_-norm below the threshold (<0.3) in the reference genome, respectively. *M* and *k* represent the total number of fragments and the number of fragments below the threshold (<0.3) in the scaffold, respectively. Scaffolds with *P*-value < 1e-005 were used as putative Y-linked scaffolds.

### Selection of putative Y-linked fragments and PCR verification

We selected a batch of fragments for further PCR verification. To improve this process, for the fragments with R_i_-norm below the threshold (<0.15), BLASTN search^23^ was performed against female-gc-assembly using default parameters^22^, fragments with the best BLAST hits (expect value < 1e-005) were discarded. The remaining putative Y-linked fragments were used for PCR amplification in eight male and eight female grass carp individuals (18 months old) randomly selected from a full-sib population. To determine whether the Y-linked sequences are specific to the wild population, we also performed PCR tests in eight male and eight female wild grass carp individuals (more than 18 months old) from different water systems. DNA was extracted from tail fins using the conventional phenol-chloroform method. The primers are listed in Supplementary Table S7. The PCR reaction system was 20 μL in volume, the PCR program was as follows: initial denaturation at 94 °C for 2 min, denaturation at 94 °C for 30 s, annealing at 51 °C for 30 s, and extension at 72 °C for 30 s. After 39 cycles, the program was extended at 72 °C for 2 min. Primers were designed using Primer5 and synthesized by Sangon Company (Shanghai, China). Y-linked sequences were defined as the occurrence of a clear amplicon or a distinct size in males but not in females.

### Y-linked genes annotation

The Y-linked scaffolds that were successfully validated by PCR tests in the full-sib population were used for Y-linked gene annotation. These scaffolds were softmasked and blasted against different databases to search for gene coding regions. Non-redundant proteins (NR), reference sequence proteins (Ref-Seq), and expressed sequence tags (EST) were downloaded from NCBI separately. Grass carp annotated genes were downloaded from the official National Center for Gene Research website (http://www.ncgr.ac.cn/grasscarp/). BLASTX was used against NR and Ref-Seq (e-value = 1e-005), BLASTN was used against the EST database and grass carp annotated genes (e-value = 1e-005). Alignments detected in at least one database were considered as Y-linked genes, which were then annotated from BLAST evidence and named according to their putative function or ‘*un-y’* for the genes without previously described functions. Finally, all Y-linked genes were blasted against the Protein Family of Domains database^44^.

### Expression analysis of four Y-linked genes

Expression experiments were performed in both male and male tissues for the Y-linked genes that lack high similarity to their female homologs. Three male and three female grass carp individuals (18 months old) were selected from the full-sib population. Brain, hypothalamus, gonad, and pituitary samples were prepared, equal quantities of the three samples from the same tissue were mixed, resulting in eight samples. RNA was isolated with Oligotex mRNA Kit (QIAGEN, Germany) in accordance with the manufacturer’s protocol. Primers were designed using Primer5 and synthesized by Sangon Company (Shanghai, China). The primers are listed in Supplementary Table S7. The PCR reaction system was 20 μL in volume, and the PCR program was as follows: initial denaturation at 94 °C for 2 min, denaturation at 94 °C for 30 s, annealing at 51 °C for 30 s, and extension at 72 °C for 30 s. After 39 cycles, the program was extended at 72 °C for 2 min. Protein sequences were aligned using ClustalW2^45^, and a Maximum Likelihood tree with Poisson correction was constructed using MEGA6^46^.

## Electronic supplementary material

Supplementary Table S1

Supplementary Table S2

Supplementary file
