# Supplementary material for: Computational identification of Y-linked markers and genes in the grass carp genome by using a pool-and-sequence method
Source: Sci Rep. 2017 Aug 15;7:8213. doi: 10.1038/s41598-017-08476-y (PMC5557828; doi:10.1038/s41598-017-08476-y)
Supplement: Supplementary file 3 — Supplementary file [file 41598_2017_8476_MOESM3_ESM.doc]

**Computational identification of Y-linked markers and genes in the grass carp genome by using a pool-and-sequence method**

Aidi Zhang ^1^, ^+^

E-mail: zhangaidi1010@gmail.com

Rong Huang ^1^, ^+^

E-mail: huangrong@ihb.ac.cn

Liangming Chen^1, 2^

E-mail: liangmingchen@126.com

Lv Xiong ^1, 2^

E-mail: 18963973548@163.com

Libo He ^1^

E-mail: helibowudi@ihb.ac.cn

Yongming Li^1^

E-mail: liyongming8080@sohu.com

Lanjie Liao^1^

E-mail: liaolj@ihb.ac.cn

Zuoyan Zhu^1^

E-mail: zyzhu@ihb.ac.cn

Yaping Wang ^1 *^

E-mail: wangyp@ihb.ac.cn

* Corresponding author

^1^ State Key Laboratory of Freshwater Ecology and Biotechnology, Institute of Hydrobiology, Chinese Academy of Sciences, Wuhan 430072, China

^2^ University of Chinese Academy of Sciences, Beijing 100049, China

^+^**These authors contributed equally to this work**

### **Table S3.** Summary of repetitive elements for male-gc-assembly and putative Y-linked scaffolds

| **Repetitive elements** | **Percentage of male-gc-assembly** | **Percentage of putative Y-linked scaffolds** |
| --- | --- | --- |
| **Retro elements** | **3.53 %** | **17.16 %** |
| SINEs | 0.12 % | 0.23 % |
| LINEs: | 1.34 % | 10.43 % |
| L2/CR1/Rex | 0.88 % | 7.08 % |
| R1/LOA/Jockey | 0.06 % | 1.07 % |
| RTE/Bov-B | 0.04 % | 0.82 % |
| L1/CIN4 | 0.17 % | 1.24 % |
| LTR elements | 2.07 % | 6.49 % |
| Gypsy/DIRS1 | 1.60 % | 5.32 % |
| Retroviral | 0.13 % | 0.66 % |
| **DNA transposons** | **12.03 %** | **10.41 %** |
| hobo-Activator | 2.10 % | 1.66 % |
| Tc1-IS630-Pogo | 1.85 % | 0.36 % |
| Tourist/Harbinger | 0.65 % | 0.81 % |
| **Satellites** | **0.86 %** | **0.49 %** |
| **Simple repeats** | **0.35 %** | **0.55 %** |
| **interspersed repeats** | **16.49 %** | **29.70 %** |
| **Low complexity** | **0.01 %** | **0.03 %** |

### **Table S4.** Inventory of the six putative Y-linked scaffolds

| Scaffolds | Gaps | Repetitive elements | | | Genes |
| --- | --- | --- | --- | --- | --- |
|  |  | Retro elements | DNA transposons | Total interspersed repeats |  |
| Sca704 | 3 | 38 (29,486 bp) | 75 (10,809 bp) | 44,524 bp | 6 |
| Sca713 | 23 | 29 (17,883 bp) | 50 (16,107 bp) | 34,899 bp | 3 |
| Sca811 | 3 | 16 (11,905 bp) | 50 (9,735 bp) | 21,867 bp | 3 |
| Sca971 | 6 | 9 (8,494 bp) | 28 (4,021 bp) | 15,532 bp | 2 |
| Sca194 | 0 | 3 (880 bp) | 5 (907 bp) | 1,787 bp | 0 |
| Sca791 | 0 | 0 | 1 (152 bp) | 0 | 0 |

### **Table S5.** Primer sequences for amplification of Y-linked sequences in the study. The first six rows correspond to Table 3 and Fig. 3. The first and last five rows correspond to Fig. 4.

| **Fragment** | **Primer^+^** | **Primer^-^** | **TM value** | **Length (bp)** |
| --- | --- | --- | --- | --- |
| Sca971_3_662 | CTTGTGTTTGTGTAAAGTGAGAGTG | TACAGGTGAAGGAATAAAATCAGTC | 52℃ | 308 |
| Sca713_52_382 | CACTAACCCTTTAAGTTGCGATAGC | AACAGCCTTTCTTCATTTGACAGAC | 54℃ | 349 |
| Sca28791_1_303 | TTCTTTTGTAATGGCTCTGATGGTC | GTGCTGGAGGCAAACACAGATT | 53℃ | 277 |
| Sca811_22_1407 | CAAATAGACCAATGCTGGAAAATGC | TATTGGTTGACTACTTGTTACAGACAC | 53℃ | 411 |
| Sca704_77_319 | ACAAATAGTGGAGCTCTGCAGCCAT | AGACTGGTTCCATCTGCACATGT | 53℃ | 292 |
| Sca971_32_446 | ACCAATAGATGAATTATTTTTCAGGC | ACATTGTTGTCTGTATGCTCTGAC | 51℃ | 479 |
| Sca971_30_188 | ACACAGGCAAAGGAGAGAAAAAAAAC | ATTAAATGTTCCACAATGAATGTAGCAAT | 52℃ | 183 |
| Sca971_9_1908 | AGAATCTGTCTCGGATGGTTAGGT | TGCCAAAAGCAAGTAAAGGGGTT | 51℃ | 350 |
| Sca971_4_1894 | ACACATGATTGTTGAGCTGAGGAG | GAAAACAACAGCAACACAGAATTACAG | 52℃ | 245 |
| Sca 971_34_3238 | AGCAGCAGGTAGCGGAAGAG | AATAACGACAGTTGACAGGATTGAATG | 52℃ | 450 |

### **Table S6.** Primer sequences for RT-PCRs in Fig. 6.

| **Gene** | **Primer+** | **Primer-** |
| --- | --- | --- |
| Sca971: *rdp-y4* | CAAGTCAAGTGTCCCCAACTAAGC | ATGGACGGTTGTTGAGGAACTG |
| Sca811: *ubq-y* | CTGCTTAATCAGTTTGTCTGGGTG | GCTCCTTTCCACTCTCTCATACAT |
| Sca971: *un-y2* | TCTGAGGTGGAAGAAGGCTG | CTGAGTATCATCGGCATAGCAGTG |
| Sca713: *un-y1* | TCTCAGAACAGTCAGAGTTTACAGG | ACAGTTTTCGGTCTGACATTGTATG |

### **Table S7.** Gene accessions that used in phylogenetic analysis of *ubq* genes showed in Fig. 7.

| **Gene name** | **Accession** | **Species** |
| --- | --- | --- |
| *ubq-y Cid* |  | *Ctenopharyngodon idellus* |
| *ubq-Female Cid* | CI000046_00461851_00464237 ^1^ | *Ctenopharyngodon idellus* |
| *ubq Srh* | XP_016405694.1 ^2^ | *Sinocyclocheilus rhinocerous* |
| *ubq Dre* | XP_692132.2 ^3^ | *Danio rerio* |
| *ubq Ame* | XP_007248082.1 ^4^ | *Astyanax mexicanus* |
| *ubq Ola* | XP_004079881.1 ^5^ | *Oryzias latipes* |
| *ubq Ddi* | XP_629545.1 ^6^ | *Dictyostelium discoideum* |
| *ubq Hum* | XP_017383462.1 | *Homo sapiens* |

### **Table S8.** The GPS coordinates of origin of the wild grass carp

| Water system | GPS coordinates |
| --- | --- |
| Zhujiang river | 113.406617, 22.751781 |
| Yangtse river | 114.141081, 30.427183 |
| Xiangjiang river | 112.934258, 28.204249 |
| Lao river | 112.446749, 29.717628 |

**Figure S1.** The workflow for identifying Y-linked sequences and genes in the grass carp genome. The figure depicts the analysis framework. First, re-sequencing of DNA pools of male and female grass carp was performed. Second, male genome assembly was fragmented and used as reference genome in mapping of pools sequencing reads. Third, the fragment-ratio method was applied to identify Y-linked sequences followed by enrichment analysis. Finally, PCR tests of Y-linked fragments and gene annotation were performed against the putative scaffolds.


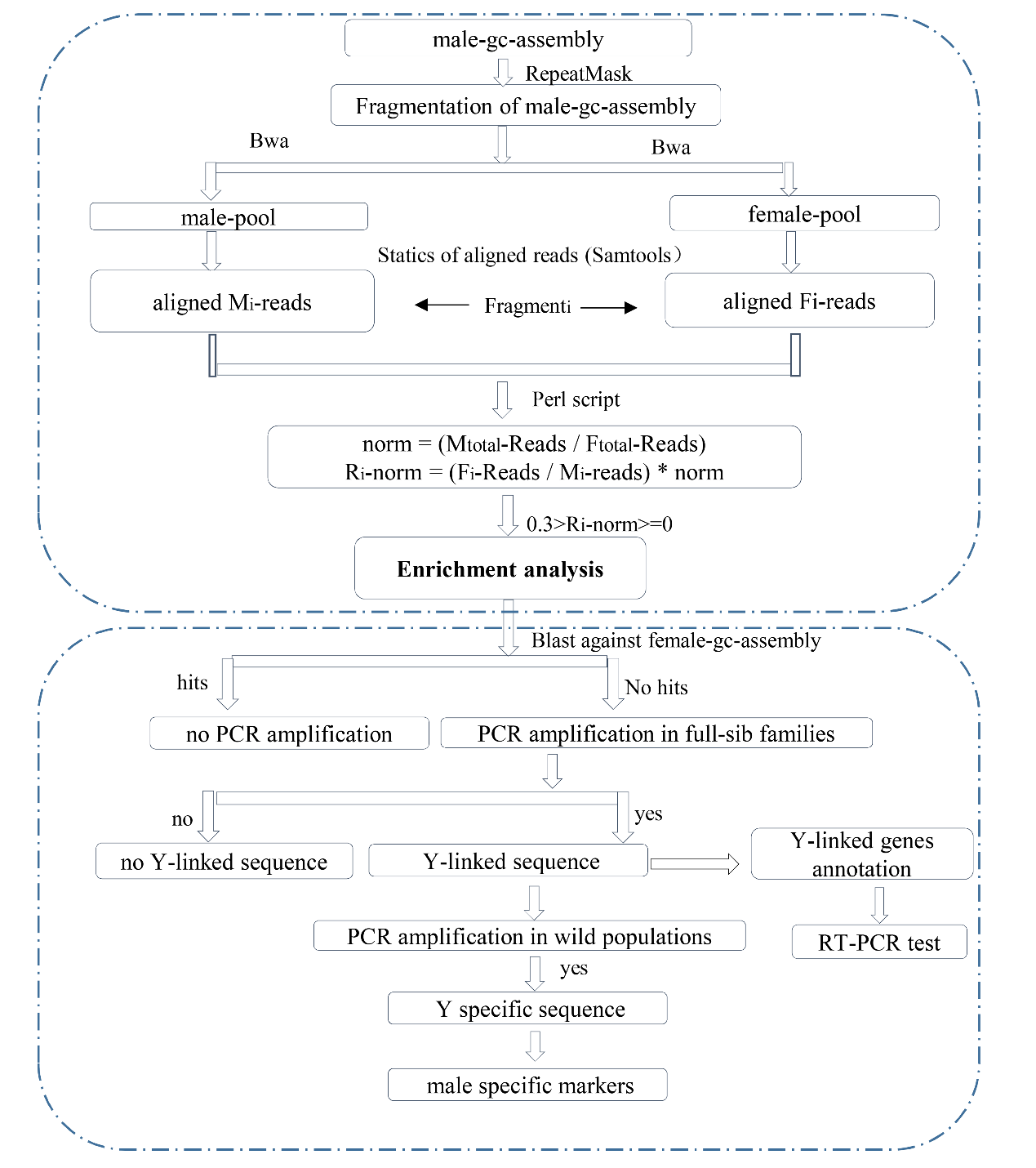


**Figure S2.** Distribution of R_i_-norm across all fragments in the grass carp genome. (a) The plot of R_i_-norm value across all fragments. The x axis represents fragments, and the y axis represents R_i_-norm value. The red dashed line indicates R_i_-norm with 1. The fragments were sorted by their corresponding R_i_-norm values. (b) The histogram of R_i_-norm value across all fragments. The x axis represents R_i_-norm value, and the y axis represents the frequency of fragments. (c) The scatter plot of R_i_-norm value across all fragments. The x axis represents fragments, and the y axis represents Ri-norm value. The yellow dashed line indicates R_i_-norm with 1, whereas the blue dashed line indicates R_i_-norm with 0.3. The fragments were sorted by their scaffold ID. These figures suggest that the R_i_ values of most fragments are close to 1.

**
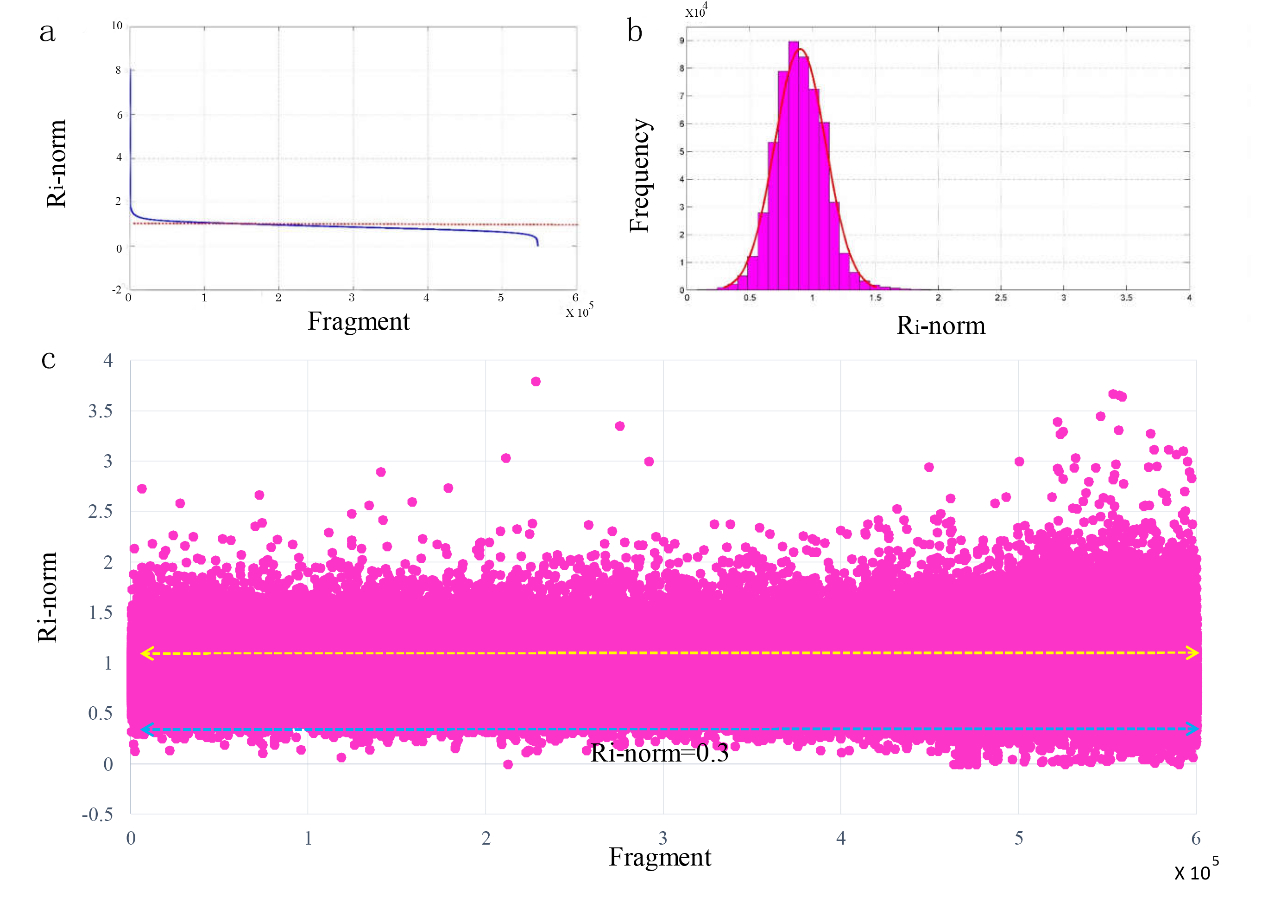
**

### **Figure S3.** PCR tests for Y-linked sequences that distributed in the four Y-linked scaffolds in wild grass carp individuals. The four Y-linked scaffolds are Sca704, Sca713, Sca28791, and Sca811. Male specificity was defined as the occurrence of a clear amplicon of a distinct size in males but not in females. The results showed that although these sequences were proved to be male specific in full-sib population, but failed to be male specific in wild samples. PCR tests for Y-linked sequences that distributed in Sca971 was shown in Fig 4


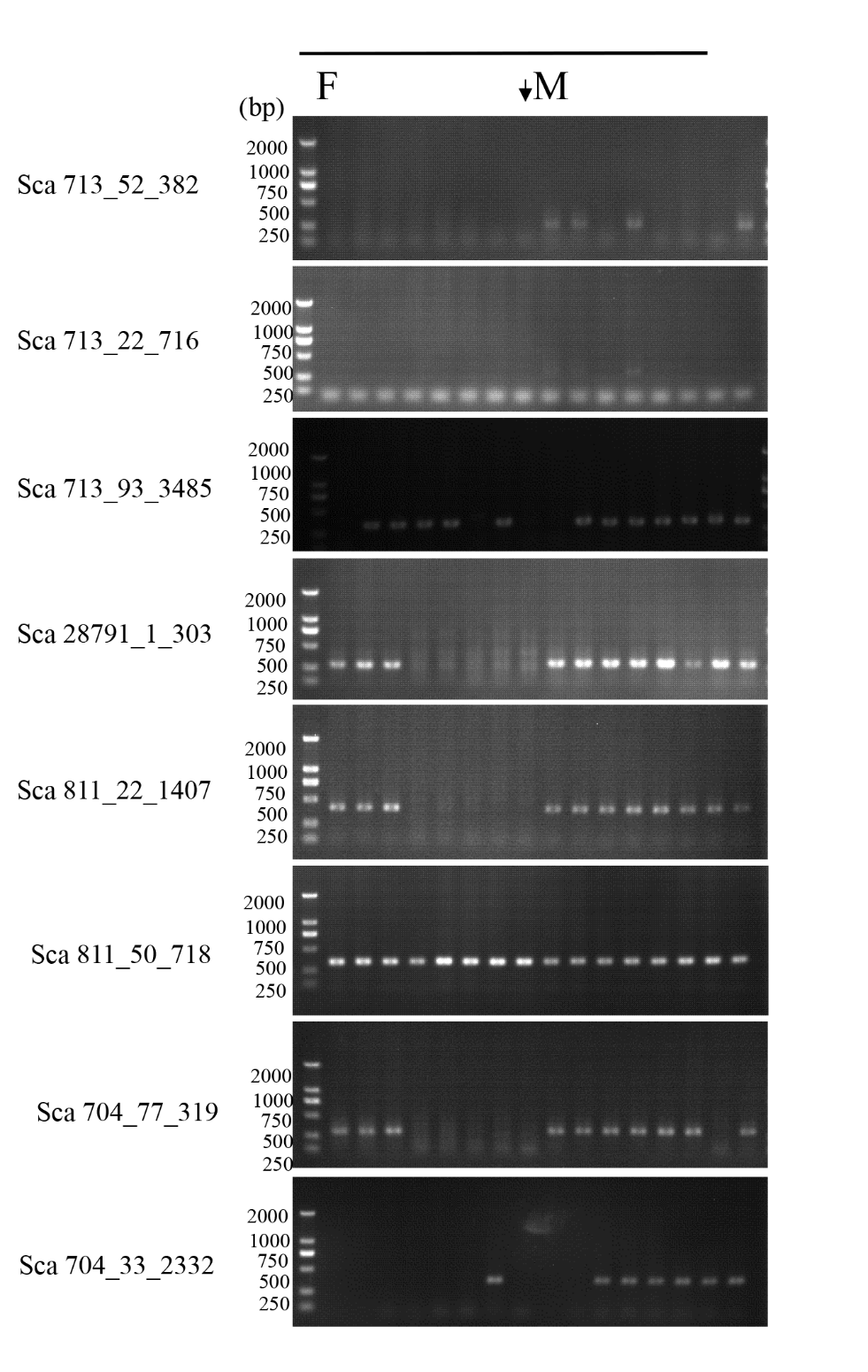


**Figure S4.** Principle of the fragment-ratio method. The fragment-ratio method allows the differentiation of Y-linked sequences from autosome and X-linked sequences. Both sexes have the same copies of autosomes. Thus, the aligned reads from the female-pool and the male-pool are roughly of the same quantity, and the R_i_-norm is close to 1. Meanwhile, the female sex has one more X chromosome than the male sex. Therefore, the aligned reads from the female-pool are twice that from the male-pool, and the R_i_-norm is close to two. Only the male sex has a Y chromosome; thus, Y-linked sequences are present only in the male-pool, and the R_i_-norm is close to 0. However, some Y-linked fragments have homologous regions to their X chromosome counterparts, leading to a few aligned reads from the female-pool, thus, the R_i_-norm is greater than 0. We set a R_i_-norm threshold of 0.3 to distinguish Y-linked sequences from the autosome and the X chromosome.


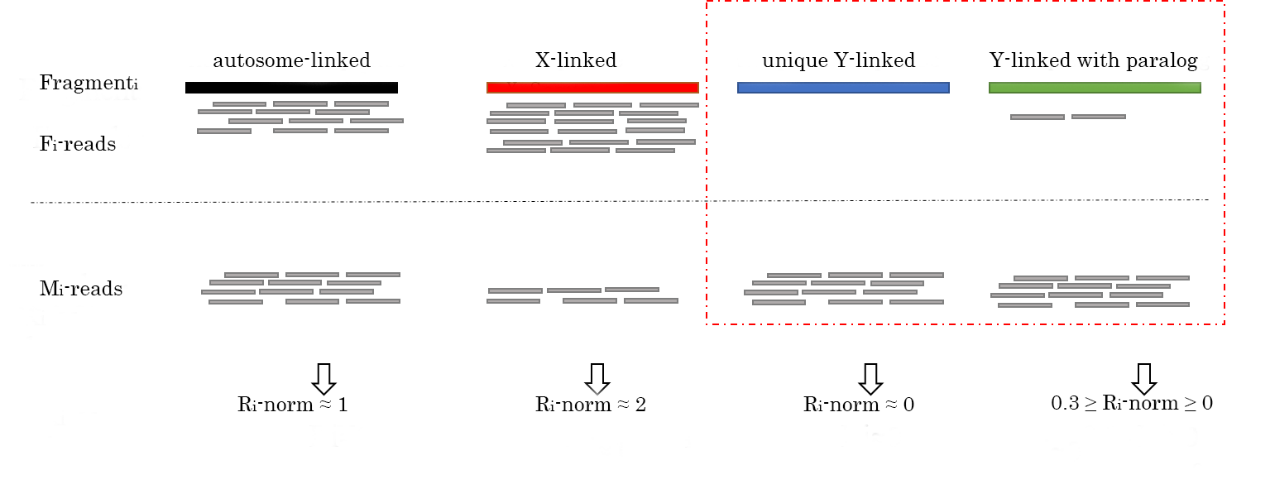


**Reference**

1 Wang, Y. et al. The draft genome of the grass carp (Ctenopharyngodon idellus) provides insights into its evolution and vegetarian adaptation. Nature genetics 47, 625-631 (2015).

2 Yang, J. et al. The Sinocyclocheilus cavefish genome provides insights into cave adaptation. BMC biology 14, 1 (2016).

3 Howe, K. et al. The zebrafish reference genome sequence and its relationship to the human genome. Nature 496, 498-503 (2013).

4 Hinaux, H. et al. Lens defects in Astyanax mexicanus Cavefish: evolution of crystallins and a role for alphaA-crystallin. Dev Neurobiol 75, 505-521 (2015).

5 Kasahara, M. et al. The medaka draft genome and insights into vertebrate genome evolution. Nature 447, 714-719 (2007).

6 Eichinger, L. et al. The genome of the social amoeba Dictyostelium discoideum. Nature 435, 43-57 (2005).
